# Enhanced Activity and Stability of Gold/Ceria-Titania for the Low-Temperature Water-Gas Shift Reaction

**DOI:** 10.3389/fchem.2019.00443

**Published:** 2019-06-14

**Authors:** James H. Carter, Parag M. Shah, Ewa Nowicka, Simon J. Freakley, David J. Morgan, Stan Golunski, Graham J. Hutchings

**Affiliations:** ^1^School of Chemistry, Cardiff Catalysis Institute, Cardiff University, Cardiff, United Kingdom; ^2^Department of Chemistry, Bath University, Bath, United Kingdom

**Keywords:** water-gas shift, gold, ceria-titania, ceria-zirconia, heterogeneous catalysis, nanoparticles

## Abstract

Gold supported on ceria-zirconia is one of the most active low temperature water-gas shift catalysts reported to date but rapid deactivation occurs under reaction conditions. In this study, ceria-titania was evaluated as an alternative catalyst support. Materials of different Ce:Ti compositions were synthesized using a sol-gel methodology and gold was supported onto these using a deposition-precipitation method. They were then investigated as catalysts for the low-temperature water-gas shift reaction. Au/Ce_0.2_Ti_0.8_O_2_ exhibited superior activity and stability to a highly active, previously reported gold catalyst supported on ceria-zirconia. High activity and stability was found to be related to the support comprising a high number of oxygen defect sites and a high specific surface area. These properties were conducive to forming a highly active catalyst with well-dispersed Au species.

## Introduction

The low-temperature water-gas shift (LTS) reaction has been extensively studied in recent years as a means of upgrading reformate by removing CO and generating H2 (Fu et al., 2003; Tibiletti et al., 2005; Reina et al., 2014). For this application, the catalyst must be both highly active and stable over long times on-stream. Current industrial catalysts based on CuZnO lack the requisite intrinsic activity at lower temperatures and this has prompted the search for new higher activity catalysts.

Au/CeO_2_ was initially reported to be highly active for the LTS reaction (Fu et al., 2001) and more recently Hardacre et al. demonstrated that the addition of Zr to the CeO_2_ support resulted in a remarkable enhancement of catalyst activity (Tibiletti et al., 2005). However, these gold-based catalysts are unstable under reaction conditions. Goguet et al. reported that the deactivation mechanism of Au/Ce_0.5_Zr_0.5_O_2_ involved a change in the morphology of the gold nanoparticle whereby the effective length of the metal-support interface, the proposed active sites, was reduced as the gold nanoparticle de-wetted (Goguet et al., 2007). We recently showed using stop-start HAADF STEM and XPS that in addition to morphological changes, particle agglomeration also occurs under comparable LTS reaction conditions at 150°C (Carter et al., 2017). The consensus is that the underlying cause of catalyst deactivation is due to a weak interaction between supported gold nanoparticles and cerium oxide-based supports.

It has been reported that defect sites in CeO_2_ can function as nucleation sites for Au and stabilize small nanoparticles of gold (Burch et al., 2010; Pojanavaraphan et al., 2013; Laguna et al., 2015). Therefore, a possible strategy to stabilize the supported Au catalyst is to synthesize a support with a high concentration of defects; one such candidate is ceria-titania. Although not as widely studied as ceria-zirconia, it has been investigated as a catalyst support for applications such as CO oxidation (Rodriguez et al., 2015; Rico-Francés et al., 2016) and dry methane reforming (Kim et al., 2015). In a similar way to ceria-zirconia, the addition of Ti to CeO_2_ gives enhanced redox properties. It should be noted that Au/TiO_2_ is not active for the LTS reaction due to the low concentration of defect sites that facilitate water activation; hence the inclusion of Ce in the many of the most active reported LTS catalysts. One of the earliest examples of ceria-titania being used as a mixed metal oxide support was by Mastelaro et al. who supported CuO on ceria-titania and reported an improvement in catalytic activity for methanol oxidation (Francisco et al., 2001). The reasoning for this was partly ascribed to the enhancement in the textural properties of the materials. Compared with the TiO_2_-only sample, the surface area of the mixed metal oxide increased while the crystallite size decreased. Manzoli et al. compared the catalytic activity of Au/CeO_2_, Au/TiO_2_, Au/Ce_0.5_Ti_0.5_O_2_, and Au/Ce_0.2_Ti_0.8_O_2_ for the low-temperature water-gas shift reaction under an idealized gas composition i.e., in the presence of just CO and H_2_O (Manzoli et al., 2007). It was shown that Au/Ce_0.2_Ti_0.8_O_2_ was the most active catalyst. Although this work highlighted the potential of ceria-titania as a support for LTS catalysts, the on-stream stability was not measured. Interestingly, Au/TiO_2_ was found to be more active than Au/CeO_2_ in contrast to other reports that did not observe LTS activity in Au/TiO_2_ below 350°C (Tibiletti et al., 2005). The difference in catalyst preparation or reaction conditions could have contributed to the discrepancies observed in the catalyst activity between these two reports.

Rodriguez et al. have made significant contributions to understanding the fundamental processes that occur in the LTS reaction using gold supported on CeO_2_/TiO_2_ as model catalysts. Recently they showed that Au/CeO_x_/TiO_2_(110) was remarkably active for LTS (Park et al., 2009). The activity of this system was markedly higher than the activity of gold supported on CeO2(111) or Au/TiO_2_(110). It was shown using a combination of density functional theory and X-ray photoelectron spectroscopy (XPS) that the Ce^3+^ cation is stabilized on CeO_x_/TiO_2_(110), leading to high activity. Rodriguez et al. also demonstrated the high activity of this support can be observed when other metals such as copper and platinum were deposited (Park et al., 2010).

In this work, a series of catalysts consisting of gold supported on a range of ceria-titanias were prepared, characterized, and investigated for the LTS reaction and compared to Au/Ce_0.5_Zr_0.5_O_2_ as well as CeO_2_. The textural and chemical properties were probed to gain an understanding of the trends in catalytic activity and stability and how these relate to the catalyst structure.

## Experimental

The Ce_1−x_Ti_x_O_2_ supports were prepared using a sol-gel methodology previously reported (Rynkowski et al., 2000). The desired ratio of Ce(NO_3_)_3_·6H_2_O (Sigma Aldrich, 99.99%) and Ti[OCH(CH_3_)_2_]_4_ (Sigma Aldrich, >97%) were dissolved in ethanol (150 cm^3^) at room temperature such that the total moles of metal in solution was 0.02. NH_4_OH (2 M, Fisher Scientific, 28–30 w/w% in H_2_O) was then added drop-wise to this solution until pH 9 was attained. The temperature of the reaction mixture was subsequently increased to 75°C for 30 min to remove the ethanol. After the majority of the solvent was removed, the mixture was filtered and the resulting solid was washed with deionised water (500 cm^3^) and dried (110°C, 16 h). The resulting powder was ground using a mortar and pestle and calcined in flowing air (450°C, 5 h, heating rate 10°C min^−1^). A range of ceria-titania (Ce_1−x_Ti_x_O_2_) materials were prepared where *x* = 0, 0.1, 0.2, 0.5, and 0.9.

Ce_0.5_Zr_0.5_O2 was prepared by a sol-gel method previously reported (Pilasombat et al., 2012). Appropriate molar quantities of Ce(NO_3_)_3_·6H_2_O and ZrO(NO_3_)_2_·xH_2_O (Sigma Alrich, 99.99%) were added to deionised water (300 cm^3^) at 80°C, under vigorous stirring in order to give the desired 1:1 molar ratio of Ce:Zr. Once the metal precursors had dissolved, NH_4_OH (0.5 M, Fisher Scientific, 28–30 w/w% in H_2_O) was added drop-wise until the pH reached 9. The reaction mixture was then immediately filtered under vacuum and washed with warm distilled water (600 cm^3^) before being dried (110°C, 16 h). The resultant solid was ground using a mortar and pestle and then calcined under flowing air at (500°C, 5 h, 10°C min^−1^).

Gold was deposited onto the supports using the deposition-precipitation method. Typically, the support was added to deionised water (200 cm^3^) at 60°C while stirring vigorously. Aqueous HAuCl_4_ (1.63 ml, 12.25 mg ml^−1^, Strem, 99.8%) was then added to give a nominal loading of 2 wt%. After 15 min, Na_2_CO_3_ (0.05 M) was added drop-wise until pH 8.0 was reached. The mixture was then stirred for 1 h before the solid was recovered by filtration under vacuum and washed with warm deionised water (600 cm^3^). The catalysts were dried (110°C, 5 h) under static air.

X-ray diffraction patterns were obtained on an X'PertPRO PANalytical instrument using Cu Kα (1.54 Å) radiation and were calibrated against a Si standard. Measurements were taken in the range of 2θ = 10−80°.

Raman spectroscopy was performed on a Renishaw ramascope using a spectrophysics 514 nm HeNe laser (20 mW). Surface area measurements were carried out at −196°C on a Quantachrome Quadrasorb SI instrument after each sample was evacuated for 2 h at 120°C. Brunauer–Emmet–Teller (BET) theory over the range P/P_0_ = 0.05–0.2 was used to calculate the specific surface area.

Scanning electron microscopy-energy dispersive x-ray spectroscopy (SEM-EDX) measurements were performed on a JEOL 6610LV equipped with an Oxford Instruments energy dispersive X-ray (EDX) analyser. The EDX instrument was calibrated using a Co standard.

XPS was carried out on a Kratos Axis Ultra-DLD XPS spectrometer equipped with an AlKα X-ray 300 W source. A C 1s reference (284.7 eV) was used as a calibration. Peaks were fitted as Gaussian Lorentzian curves GL(30) using CasaXPS software.

The actual Au loadings of the catalysts were determined using an Agilent 4,100 MP-AES spectrometer equipped with a nitrogen plasma. A sample of each catalyst (50 mg) was digested in aqua regia (4 cm^3^) at ambient temperature for 16 h, before dilution in deionized water up to a total volume of 25 cm^3^. Remaining solids were filtered before analyzing the final solution.

Water-gas shift catalysis was carried out on a custom-made fixed-bed flow reactor at a temperature of 150°C. The catalyst (0.150 g) was suspended between two pieces of glass wool. The gas feed consisted of 2% CO, 2% CO_2_, 7.5% H_2_O, 8.1% H_2_, and N_2_ to balance. The total flow rate was 100 ml min^−1^ which corresponds to a GHSV of 52,000 h^−1^).

## Results and Discussion

**Figure 1:**
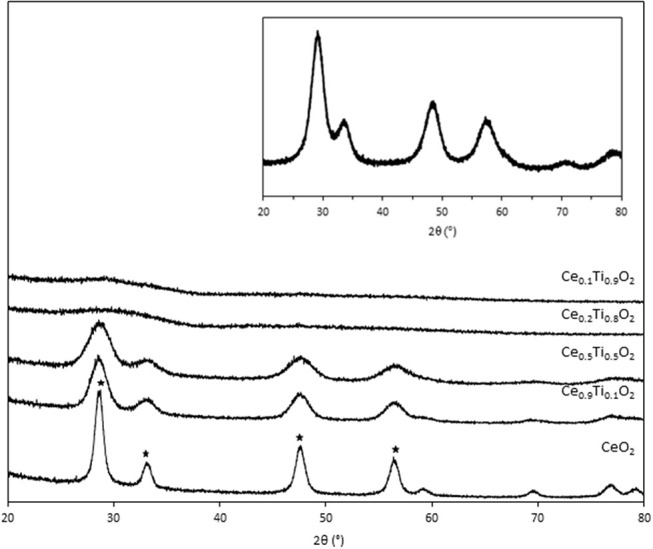
XRD stack of synthesized the Ce_x_Ti_1−x_O_2_ materials, showing cubic fluorite reflection of CeO_2_ (*). Inset: XRD pattern of Ce_0.5_Zr_0.5_O_2_.

Initially, the textural, physical, and chemical properties of the Ce_1−x_Ti_x_O_2_ supports were measured and compared to Ce_0.5_Zr_0.5_O_2_. It was previously established that the optimum Ce:Zr molar ratio for gold-catalyzed LTS was 1:1 (Pilasombat et al., 2012), therefore Ce_0.5_Zr_0.5_O_2_ was used as the benchmark catalyst support. The bulk elemental composition of prepared supports was measured using SEM-EDX and is summarized in Table 1. The measured Ce:Ti ratios were close to that of the nominal values, indicating the efficacy of the preparation method. The XRD patterns are shown in Figure 1 and feature significant differences across the range of Ce_1−x_Ti_x_O_2_ materials prepared. CeO_2_ exhibits several reflections: at 28.7, 33.2, 47.6, and 56.5°, which, respectively, correspond to the (111), (200), (220), and (311) planes of the fluorite-type cubic structure (Rico-Francés et al., 2016). These features decrease in intensity as Ti content increases, indicating a loss in the long-range order of the material, which has previously been observed and ascribed the formation of a solid solution. Specifically, Ce_0.9_Ti_0.1_O_2_ and Ce_0.5_Ti_0.5_O_2_ exhibited a fluorite structure, while Ce_0.2_Ti_0.8_O_2_ and Ce_0.1_Ti_0.9_O_2_ were amorphous. The Scherrer equation was used to estimate the crystallite size of the support materials from the most intense reflection, shown in Table 1. The estimated crystallite size of CeO_2_ was 123 Å. The introduction of 10 mol % Ti to CeO_2_ induced a reduction in the crystallite size to 50 Å. Reduced crystallite sizes were also observed in the Ce_0.2_Ti_0.8_O_2_ and Ce_0.5_Ti_0.5_O_2_ materials while the crystallite size of CeZrO_4_ was estimated to be 70 Å.

**Table 1:** Textural and chemical properties of the Ce_x_Ti_1−x_O_2_ and Ce_0.5_Zr_0.5_O_2_ catalysts.

**Sample**	**Ce:M molar ratio**		**Specific surface area^b^ (m^**2**^ g^**−1**^)**	**Crystallite size^c^ (Å)**
	**Nominal**	**Actual^a^**		
CeO_2_	–	–	62	123
Ce_0.9_Ti_0.1_O_2_	9	10.2	106	51
Ce_0.5_Ti_0.5_O_2_	1	0.94	143	42
Ce_0.2_Ti_0.8_O_2_	0.25	0.23	300	amorphous
Ce_0.1_Ti_0.9_O_2_	0.11	0.11	191	amorphous
Ce_0.5_Zr_0.5_O_2_	1	1.23	83	71

a *Elemental composition determined using SEM-EDX*.

b *Specific surface area measurements determined using N_2_ porosimetry BET method*.

c *Crystallite size determined using the Scherrer Equation from XRD patterns of each material*.

N_2_ physisorption was carried out to measure the specific surface area of the support materials. The BET surface area measurements are presented in Table 1. The specific surface area of CeO_2_ was measured to be 62 m^2^ g^−1^, respectively, while the mixed metal oxides all exhibited higher surface areas, consistent with the lower crystallite size determined using the Scherrer equation. The highest surface areas were observed in the amorphous materials, with Ce_0.2_Ti_0.8_O_2_ exhibiting a specific surface area of 300 m^2^ g^−1^ followed by Ce_0.1_Ti_0.9_O_2_, which was measured to be 190 m^2^ g^−1^. Ce_0.5_Zr_0.5_O_2_ exhibited a surface area of 83 m^2^ g^−1^, lower than the Ce_1−x_Ti_x_O_2_ materials.

**Figure 2:**
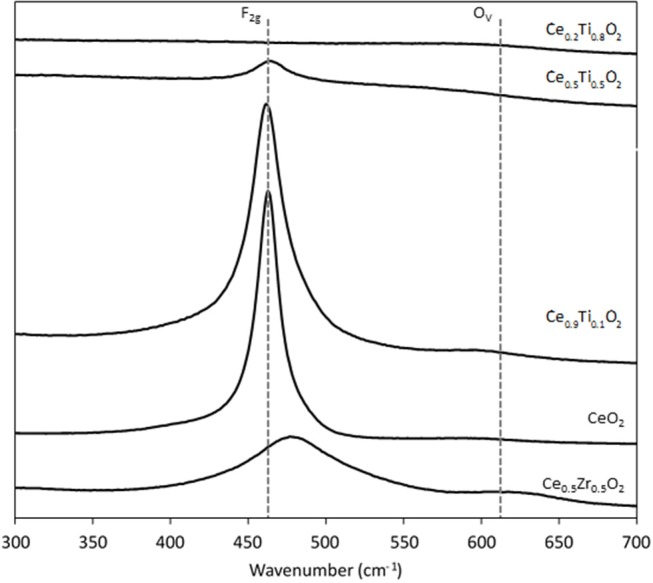
Raman spectra of the synthesized Ce_0.5_Zr_0.5_O_2_ and the Ce_x_Ti_1−x_O_2_ supports, showing the F_2g_ mode and the O_v_ modes.

**Figure 3:**
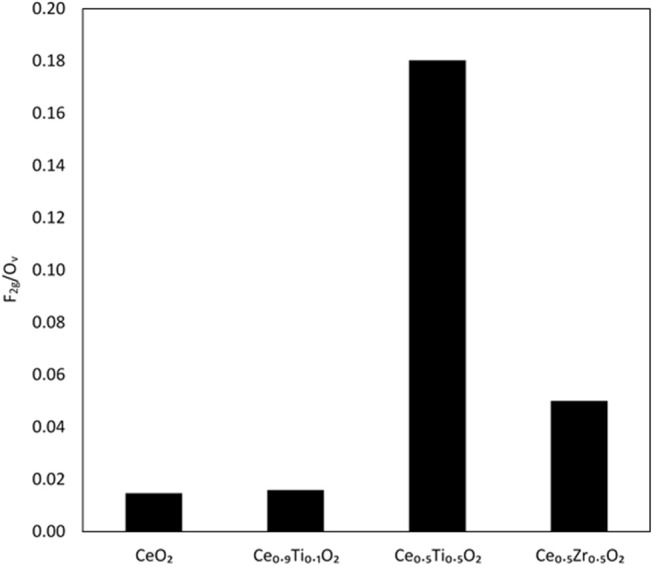
Comparison of oxygen vacancies on Ce-based oxides as determined from Raman spectroscopy, using the ratio of the F_2g_ mode and the O_v_ mode.

Each support was examined using Raman spectroscopy and the resulting spectra are presented in Figure 2. There are two main features in the prepared materials: a large band at 460 cm^−1^, associated with the F_2g_ stretching mode of the CeO_2_ fluorite structure and a weak mode at 600 cm^−1^, denoted O_v_ and assigned to oxygen defects on the surface of the cubic structure of CeO_2_ (Graham et al., 1991; Spanier et al., 2001; Laguna et al., 2010, 2015). The line shape and position of the F_2g_ mode varies across the Ce_1−x_Ti_x_O_2_ supports, as previously reported. Spanier et al. reported that the size of a CeO_2_ nanoparticle affected both the position of the band as well as the line shape, with smaller particles giving broader, more asymmetric bands at lower energies (Spanier et al., 2001). In addition, the intensity of the band has been shown to be proportional to the particle size of CeO_2_ (Graham et al., 1991). As Ti content increases, the line shape of this Raman feature decreases in intensity and broadens, suggesting that the particle size decreased after the addition of Ti. This observation is consistent with the BET and XRD data that showed an increase in surface area and a decrease in crystallite size, respectively. The relative concentration of oxygen vacancies in each sample was calculated using a method previously described that involves the calculation of the ratio of the area of the O_v_ mode to the F_2g_ mode (Graham et al., 1991; Pu et al., 2007; Laguna et al., 2010). The calculated ratios are shown in Figure 3 for the samples that exhibited an F_2g_ mode and show that an increase in Ti content leads to a significant increase in the O_v_/F_2g_ ratio. Ce_0.5_Ti_0.5_O_2_ exhibited the highest ratio, but Ce_0.2_Ti_0.8_O_2_ did not exhibit a measurable O_v_ mode, therefore calculating the ratio was not possible. However, the previous textural characterization of Ce_0.2_Ti_0.8_O_2_ suggests that at this high surface area, small crystallite material would possess a high number of defect sites. Ce_0.5_Zr_0.5_O_2_ was included for comparison, as this material is known to have a high concentration of defects. These data demonstrate that the introduction of Ti to CeO_2_ causes a large increase in the concentration of oxygen defects in the mixed metal oxides. The importance of oxygen defects in supported metal catalysts has been previously reported by Laguna et al. (2015) who showed that oxygen defect sites promote high dispersion and strong anchoring of gold on the support. In the context of the LTS reaction, the oxygen vacancies serve as activation sites for water and are therefore required in abundance for highly active LTS catalysts. The data presented in Figure 3 indicate that Ce_0.5_Ti_0.5_O_2_ (and Ce_0.2_Ti_0.8_O_2_) have a higher concentration of oxygen defects than Ce_0.5_Zr_0.5_O_2_, suggesting that these materials would be effective catalyst supports for the LTS reaction. Characterization of the physical and chemical properties of the mixed cerium-titanium oxides showed that high surface area materials with a high density of defect sites could be prepared using the sol-gel methodology.

Gold was deposited onto the prepared supports using a deposition-precipitation (DP) method previously reported (Carter et al., 2016). It is common for catalysts prepared using DP to have metal loadings lower than their nominal value due to the poor interaction between aqueous metal precursor and support surface that enhances metal dispersion. The actual metal loadings of the catalysts were determined using MP-AES after digestion of the catalysts in *aqua regia*. The supported metal loading is shown in Table 2. The measured values show that the Au loading increases with Ce content. Au/CeO_2_ had a metal loading of 1.72 wt% while Au/Ce_0.1_Ti_0.9_O_2_ had 0.69 wt% Au. These data indicate that the surface charge of TiO_2_ is more negative at pH 8 than that of CeO_2_, consistent with previously reported PZC values (De Faria and Trasatti, 1994; Kosmulski, 2002).

**Table 2:** Analysis of the 2 wt% Au/Ce_x_Ti_1−x_O_2_ and 2 wt% Au/Ce_0.5_Zr_0.5_O_2_ catalysts.

**Catalyst**	**Metal loading^a^ (%)**	**Ce:M molar ratio^b^**	**Au species composition (%)^b^**			**Binding energy (eV)^b^**		
			**Au^**0**^**	Au^0^*	**Au^**3+**^**	**Au^**0**^**	Au^0^*	**Au^**3+**^**
**Au/CeO**_**2**_	1.72	–	63.0	26.7	10.3	83.6	85.1	86.9
**Au/Ce**_**0.9**_**Ti**_**0.1**_**O**_**2**_	1.70	10.5	68.3	10.7	21.0	84.3	85.7	86.8
**Au/Ce**_**0.5**_**Ti**_**0.5**_**O**_**2**_	1.30	0.7	58.0	24.9	17.2	84.1	85.2	86.9
**Au/Ce**_**0.2**_**Ti**_**0.8**_**O**_**2**_	1.08	0.9	50.6	28.2	21.1	84.1	85.2	86.9
**Au/Ce**_**0.1**_**Ti**_**0.9**_**O**_**2**_	0.69	0.1	75.1	24.9	0	84.2	85.5	-
**Au/Ce**_**0.5**_**Zr**_**0.5**_**O**_**2**_	1.59	1.8	61.4	20.8	17.8	84.0	85.4	86.9

a *Total metal loading determined using MP-AES*.

b *Ce:M molar ratio (where M = Ti or Zr) and Au species composition determined using XPS*.

**Figure 4:**
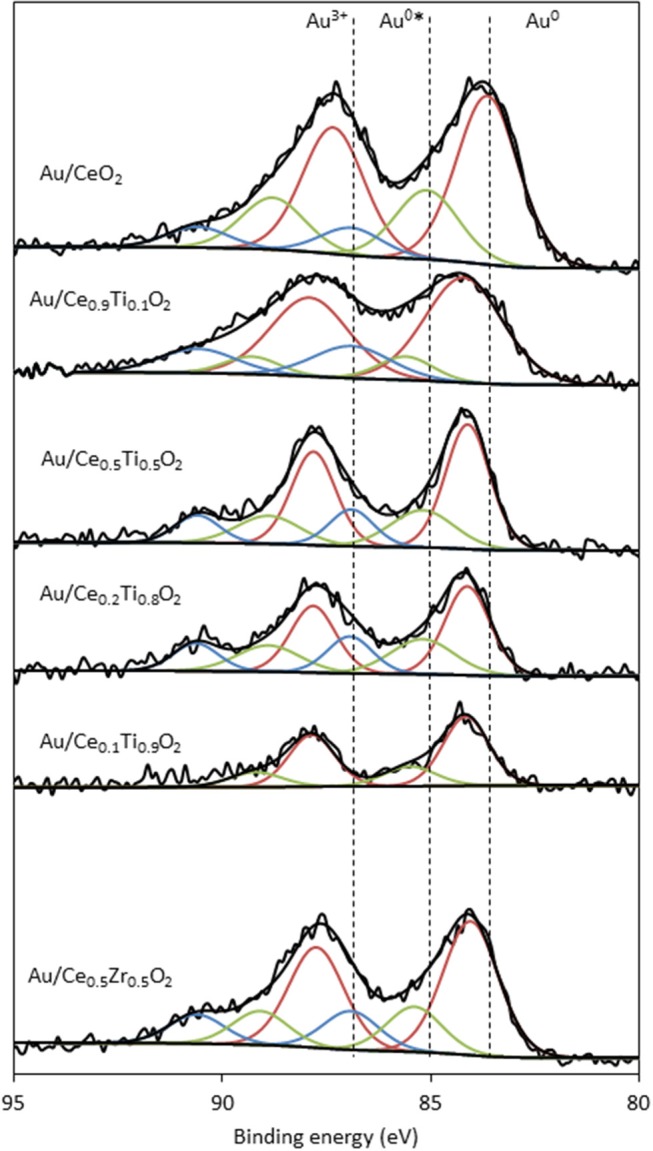
XPS analysis of the Au 4f spectra of supported gold catalysts showing the identification of three Au species: Au^0^ (red), Au^0^^*^ (green) and Au^3+^ (blue).

XPS analysis of the prepared catalysts was carried out to examine the composition of the catalyst surface. Comparison of the Ce:M molar ratio using XPS analysis allows the comparison between the surface composition and the bulk composition, determined using SEM-EDX. The values match the EDX values from Table 1 closely, with the exception of Ce_0.2_Ti_0.8_O_2_, which has a higher than expected Ce:Ti ratio from the XPS analysis. This suggests some surface enrichment of Ce in the catalyst support. The deconvoluted Au 4f spectrum of each catalyst is presented in Figure 4. Three distinct Au species were observed in each catalyst. In Au/Ce_0.5_Zr_0.5_O_2_, these were measured to be at 84.0, 85.4 and 86.9 eV, which were assigned to Au^0^, Au^0^^*^, and Au^3+^. The notation Au^0^^*^ refers to small metallic Au nanoparticles (i.e., below ~2 nm) and has been widely reported (Luo et al., 2001; Willneff et al., 2006; Rodriguez et al., 2014; Zhou et al., 2015; Carter et al., 2016). It was also noted that the binding energy of each Au species was dependent on the composition of the support, consistent with previous investigations (Wayne Goodman, 2010). The composition of different Au species in each catalyst sample varied systematically across the sample set as shown in Table 2. Of particular importance are the Au^3+^ and Au^0^^*^ species, which were previously identified as being catalytically active or precursors to catalytically active species (Burch et al., 2010; Carter et al., 2017). Au/Ce_0.2_Ti_0.8_)_2_ exhibited the highest concentration of such Au species, suggesting that it would exhibit the highest LTS activity.

**Figure 5:**
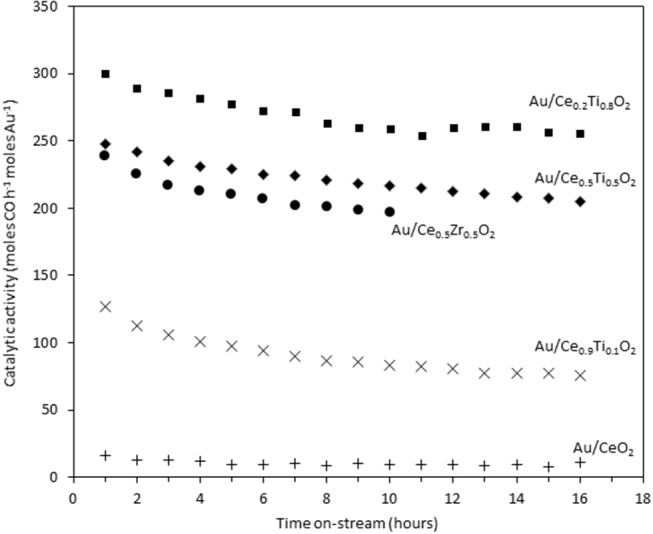
Time on-stream activity plots for gold catalysts 10 the LTS reaction: 0.150 g catalyst, 150°C, 100 ml min^−1^, 2% CO, 2% CO_2_, 7.5% H_2_O, 8.1% H_2_, 80.4% N_2_.

**Figure 6:**
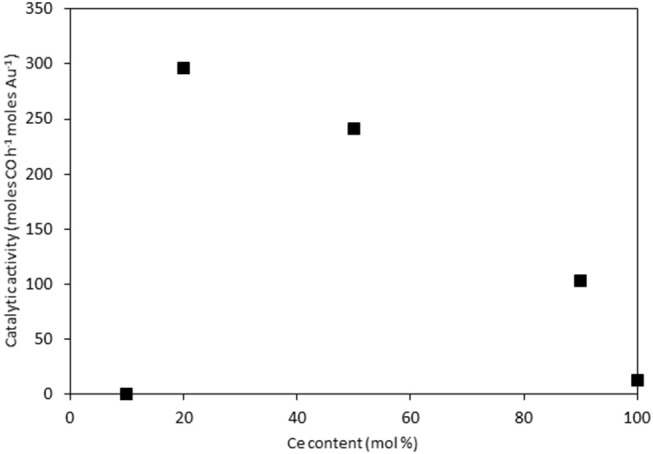
LTS activity for Au/Ce_x_Ti_1−x_O_2_ catalysts after 90 min on-stream: 0.150 g catalyst, 150°C, 100 ml min^−1^, 2% CO, 2% CO_2_, 7.5% H_2_O, 8.1% H_2_, 80.4%N_2_.

**Figure 7:**
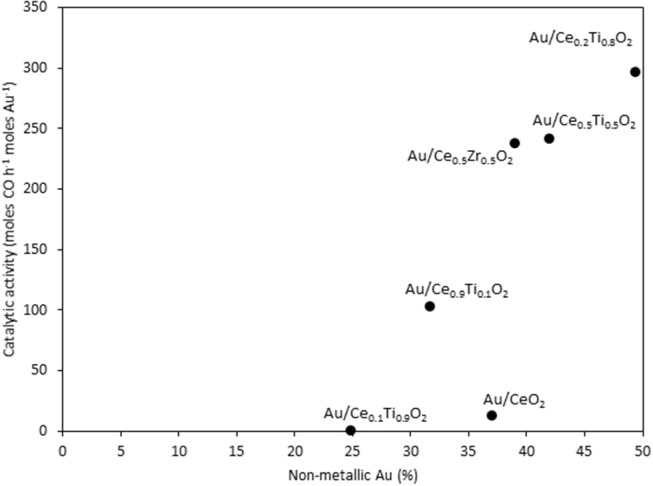
The LTS activity after 1 h on-stream plotted against the concentration of non-metallic Au in the sample, as determined by XPS.

The catalysts were investigated for their activity in the LTS reaction 150°C. The time on-line data is shown in Figure 5. The units of catalytic activity are expressed as the number of moles of CO converted per hour, per mole of gold (moles CO h^−1^ moles Au^−1^) to account for the variation in the metal loadings between each catalyst, which is shown in Table 2. It should be noted that the conversions reported herein are far from the thermodynamic equilibrium conversion, which was calculated to be > 99% (the most active catalysts in this work gave approximately 50% conversion). The order of activity was: Au/Ce_0.2_Ti_0.8_O_2_ > Au/Ce_0.5_Ti_0.5_O_2_ > Au/Ce_0.5_Zr_0.5_O_2_ > Au/Ce_0.9_Ti_0.1_O_2_ > Au/CeO_2_. It was previously reported that Au/Ce_0.2_Ti_0.8_O_2_ was more active than Au/Ce_0.5_Ti_0.5_O_2_ (Manzoli et al., 2007), which is consistent with the current work and could reflect the difference in the number of active sites that can be stabilized on the mixed metal oxide at different Ce:Ti ratios. Au/Ce_0.1_Ti_0.9_O_2_ did not exhibit catalytic activity. It is likely that Au/Ce_0.1_Ti_0.9_O_2_ was not active for the same reason that TiO_2_ is not active under similar conditions; that water cannot be efficiently activated on the surface (Tibiletti et al., 2005). Au/Ce_0.2_Ti_0.8_O_2_ and Au/Ce_0.5_Ti_0.5_O_2_ exhibited higher activity than the benchmark Au/Ce_0.5_Zr_0.5_O_2_catalyst, demonstrating the effectiveness of ceria-titania mixed metal oxides as catalyst supports and their proficiency to stabilize small gold nanoparticles, the active species in the LTS reaction (Fu et al., 2003; Tibiletti et al., 2005). The high dispersion of Au in these catalysts is inferred from the distribution of Au species measured by XPS. The most active catalysts featured a high proportion of Au^3+^ or Au^0^^*^. Comparison of the ceria-titania catalysts is shown in Figure 6, which plots the activity after 90 min on-stream against the Ce content (mol %) in the mixed metal oxide supports. It is clear that a combination of cerium and titanium is required for a highly active catalyst. The origin of this synergy is likely due to two properties of the support: surface area and oxygen defect density. A high surface area can enable a high dispersion of supported metal, while a high oxygen defect density gives a catalyst with many nucleation sites for gold and activation sites for water (Pojanavaraphan et al., 2013; Laguna et al., 2015). Figure 7 shows the relationship between catalytic activity and the proportion of non-metallic Au in the sample, demonstrating a positive correlation. Au/CeO_2_ is a slight outlier in this trend, but the CeO_2_ support was found to have relatively few oxygen defect sites, which means the catalytic activity may be limited by the activation of water.

**Figure 8:**
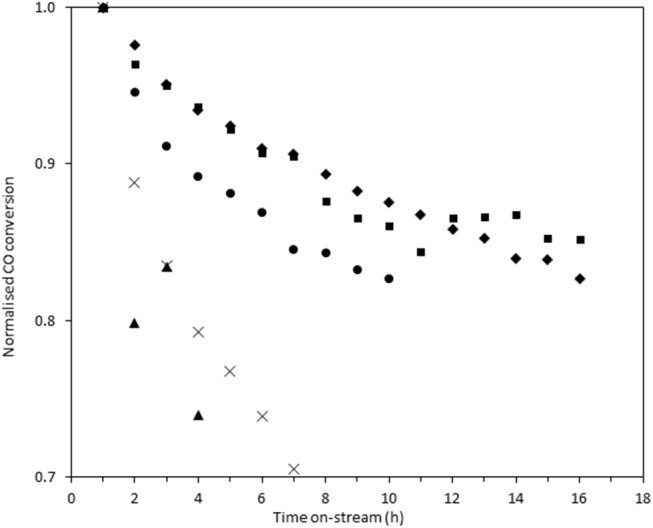
Normalized on-stream conversion of Au/Ce_0.2_Ti_0.8_O_2_ (■), Au/Ce_0.5_Ti_0.5_O_2_(♦), Au/Ce_0.5_Zr_0.5_O_2_ (•), Au/Ce_0.9_TI_0.1_O_2_ (**×**), and Au/CeO_2_ (▴) in LTS reaction 0.150 g catalyst, 150°C, 100 ml min^−1^, 2% CO, 2% CO_2_, 7.5% H_2_O, 8.1% H_2_ 80.4% N_2_. Conversions were normalized against their conversion at 1 h on-stream.

In order to measure the stability of the catalysts, the time on-line data was plotted as the normalized conversion, as described in the experimental section (Figure 8). The rate of deactivation varies significantly between the catalysts over the time period investigated. The most stable catalysts were Au/Ce_0.5_Ti_0.5_O_2_ and Au/Ce_0.2_Ti_0.8_O_2_, which retained 87 and 88% of their initial activity after 10 h on-stream. The benchmark catalyst, Au/Ce_0.5_Zr_0.5_O_2_, retained 83% of its activity after 10 h. While these catalysts were more stable, the improvement represents a marginal increase in stability but in the case of Au/Ce_0.2_Ti_0.8_O_2_, the catalyst exhibited enhanced stability in a more active form. Based on the consensus that the deactivation of Au/Ce_0.5_Zr_0.5_O_2_ in the LTS reaction is due to the metal-support interaction, the enhanced stability observed in the ceria-titania materials indicates that this interaction is stronger than in the ceria-zirconia catalyst. The origin of the stabilizing effect is likely due to the defect-rich support that efficiently stabilizes active Au species. In the case of Ce_0.2_Ti_0.8_O_2_, a high population of active Au species were stabilized on the support, producing the most active and stable catalyst. These data therefore demonstrate that other Ce-based mixed metal oxides should be considered as catalyst supports and that highly defective oxidic supports can stabilize highly-dispersed metal species. High-resolution electron microscopy is typically used to image supported metal clusters and atoms, however the difficulty in obtaining sufficient mass contrast on CeO_2_ based supported metal catalysts to resolve sub-nm species is well-known(Guo et al., 2016; Carter et al., 2017; Stere et al., 2017). In this case, despite using aberration-corrected scanning transmission electron microscopy, it was not possible to resolve these species. Consequently, statistically-relevant particle size distributions in the fresh and used catalysts could not be obtained.

## Conclusions

A series of mixed metal oxides formed of cerium and titanium were prepared using a sol-gel methodology. These were compared to Ce_0.5_Zr_0.5_O_2_, the benchmark support for gold-catalyzed LTS catalysts. The textural and chemical properties of the Ce_x_Ti_1−x_O_2_ materials were measured and it was found that the Ce:Ti ratio was a crucial factor in determining these properties. The highest surface area was displayed by amorphous Ce_0.2_Ti_0.8_O_2_. After the subsequent deposition of Au onto the supports, the catalysts were characterized using XPS. The most Au/Ce_x_Ti_1−x_O_2_ catalysts showed a high abundance of Au species typically assigned to highly dispersed non-metallic gold species. During extended LTS testing, Au/Ce_0.2_Ti_0.8_O_2_ and Au/Ce_0.5_Ti_0.5_O_2_ exhibited higher activity and stability than Au/Ce_0.5_Zr_0.5_O_2_. The enhanced activity was ascribed to the high density of oxygen vacancies present on the ceria-titania, which is known to stabilize small gold species. These findings demonstrate that improvements to catalyst stability can be made with careful consideration of the necessary properties to achieve highly dispersed, well-anchored gold species.

## Data Availability

The datasets generated for this study are available on request to the corresponding author.

## Author Contributions

JC, EN, SF, SG, and GH designed the experiments. JC and PS carried out catalyst synthesis. JC carried out catalyst testing. DM carried out and analyzed XPS. JC carried out XRD, surface area measurements, MP-AES, EDX, and Raman spectroscopy. JC and GH wrote the manuscript. SG and GH directed the research.

### Conflict of Interest Statement

The authors declare that the research was conducted in the absence of any commercial or financial relationships that could be construed as a potential conflict of interest.
